# A combined biomarker panel shows improved sensitivity and specificity for detection of ovarian cancer

**DOI:** 10.1002/jcla.24232

**Published:** 2022-01-07

**Authors:** Lu Mao, Yong Tang, Ming‐jing Deng, Chun‐tao Huang, Dong Lan, Wen‐Zheng Nong, Li Li, Qi Wang

**Affiliations:** ^1^ Guangxi Medical University Cancer Hospital Nanning China; ^2^ Wuming Hospital of Guangxi Medical University Nanning China; ^3^ Institute of Life Sciences Guangxi Medical University Nanning China; ^4^ The First Affiliated Hospital of Guangxi Medical University Nanning China; ^5^ National Hospital of Guangxi Zhuang Autonomous Region Nanning China; ^6^ Key Laboratory of Early Prevention and Treatment for Regional High Frequency Tumor Ministry of Education Nanning China

**Keywords:** biomarker, CCL18, combined serum detection, diagnosis, ovarian cancer, prognosis

## Abstract

**Background:**

Combined biomarkers can improve the sensitivity and specificity of ovarian cancer (OC) diagnosis and effectively predict patient prognosis. This study explored the diagnostic and prognostic values of serum CCL18 and CXCL1 antigens combined with C1D, FXR1, ZNF573, and TM4SF1 autoantibodies in OC.

**Methods:**

CCL18 and CXCL1 monoclonal antibodies and C1D, FXR1, ZNF573, and TM4SF1 antigens were coated with microspheres. Logistic regression was used to construct a serum antigen‐antibody combined detection model; receiver‐operating characteristic curve (ROC) was used to evaluate the diagnostic efficacy of the model; and the Kaplan‐Meier method and Cox regression models were used for survival analysis to evaluate the prognosis of OC. Data from The Cancer Genome Atlas (TCGA) and Genotype‐Tissue Expression (GTEx) projects and online survival analysis tools were used to evaluate prognostic genes for OC. The CIBERSORT immune score was used to explore the factors influencing prognosis and their relationship with tumor‐infiltrating immune cells.

**Results:**

The levels of each index in the blood samples of patients with OC were higher than those of the other groups. The combined detection model has higher specificity and sensitivity in the diagnosis of OC, and its diagnostic efficiency is better than that of CA125 alone and diagnosing other malignant tumors. CCL18 and TM4SF1 may be factors affecting the prognosis of OC, and CCL18 may be related to immune‐infiltrating cells.

**Conclusions:**

The serum antigen‐antibody combined detection model established in this study has high sensitivity and specificity for the diagnosis of OC.

## Graphical abstract

CCL18 and CXCL1 monoclonal antibodies, and C1D, FXR1, ZNF573, and TM4SF1 antigens were coated with microspheres. Biotinylated CCL18 and CXCL1 polyclonal antibodies, and C1D, TM4SF1, FXR1, and ZNF573 goat anti‐IgG polyclonal antibodies were used as detection antibodies. The red and green dual‐color laser is employed to detect red classification fluorescence on microspheres and green reporter fluorescence on the reporter molecule, allowing for the determination of the content of each index in the serum sample. One hundred fifty cases of ovarian cancer, 150 cases of benign tumors and 100 cases of healthy women were used to construct a combined detection model, and another 290 samples were used for clinical verification. The results showed that the combined detection model had higher specificity and sensitivity in the diagnosis of ovarian cancer. The diagnostic efficiency of ovarian cancer is better than other cancers, and it is better than CA125 alone in the diagnosis.

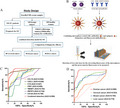

## 1 INTRODUCTION

Ovarian cancer (OC) is one of the most frequent malignant diseases that seriously threatens women's life and health. Its incidence ranks third among malignant tumors of the female reproductive tract.^1^ However, the mortality rate of OC ranks first.^2^ In 2019, there were about 21,750 new cases of OC in the United States, with 13,940 deaths and a mortality rate exceeding 60%.^3^ Due to the hidden incidence of OC, the lack of typical clinical symptoms, and early diagnosis methods, about 70% of OC patients are already in the middle and advanced stages when diagnosed.^4^, ^5^, ^6^ Among the current technical approaches for non‐invasive diagnosis of OC, a pelvic examination is not sufficiently sensitive to detect ovarian masses, and the level of serum tumor marker CA125 is elevated in 90% of patients with advanced disease, but in only 50% of patients with stage I tumors.^5^ Therefore, improving the early detection rate of OC and screening out factors that influence the prognosis of OC is critical. At present, no suitable biomarkers that can be used for early diagnosis, curative effect detection, and prognostic assessment of OC have been identified.^7^, ^8^, ^9^, ^10^, ^11^ Current studies have shown that combining multiple biomarkers can not only improve the sensitivity and specificity of early diagnosis of OC but also predict the choice of effective treatment methods and prognosis.^12^, ^13^, ^14^

With the progress of tumor immunotherapy, the correlation between immunity and the tumor has received considerable attention. The level of immune cell infiltration in the tumor is associated with tumor growth, progression, and patient outcome and has become the focus of research in recent years. Some scholars have proposed a method to calculate the composition of immune cells from the gene expression profile of complex tissues. This method has been verified by flow cytometry in colorectal cancer, lung cancer, and follicular lymphoma and can be used in large‐scale analysis of gene expression profiles.^15^, ^16^

Serological analysis of recombinantly expressed cDNA clone (SEREX) technology was performed to identify serum IgG autoantibodies C1D, TM4SF1, FXR1, and ZNF573 that are significant for the diagnosis of OC,^17^ and the surface‐enhanced laser desorption/ionization‐time of flight mass spectrometry (SELDI‐TOF‐MS) technology was used to identify serum antigens CCL18 and CXCL1 for OC.^18^ Through the application of a liquid suspension chip detection system, a combined detection method of serum CCL18 and CXCL1 antigens and C1D, TM4SF1, FXR1, and ZNF573IgG autoantibodies for the diagnosis of OC was successfully established. Its sensitivity and specificity were higher compared with the conventional enzyme‐linked immunosorbent assay (ELISA) method.^19^, ^20^ It was concluded that the diagnostic model with combined detection of serum antigen and antibody is better than serum CA125 in the diagnosis of OC.^21^ A model for diagnosing ovarian malignant tumors was successfully constructed and validated by conducting preliminary experiments. However, no effective indicators were found to predict the survival and prognosis of OC. In addition, because the sample size and the number of samples included in the study were not effective, and there were fewer samples from other malignant tumor groups, we expanded the sample size and the types of malignant tumor control groups. The combined detection model of serum antigen and antibody was used to diagnose OC and verify other malignant tumors to compare their diagnostic efficiency. Moreover, the overall survival (OS) of patients with OC was evaluated. The differences in the expression of the six genes in normal ovarian tissue and OC tissue were analyzed based on the Cancer Genome Atlas (TCGA) and the Genotype‐Tissue Expression (GTEx) databases. The relationship between the expression of these genes in OC tissues and the OS time was also evaluated. Finally, the immune score of the CIBERSORT algorithm was used to further analyze the relationship between the established prognostic indicators and infiltrating immune cells.

## 2 METHODS

### 2.1 Case selection and data sources

Serum samples were obtained from 740 patients diagnosed pathologically from January 2014 to January 2021 at the Guangxi Medical University Cancer Hospital, 100 women with no abnormal physical examination from the Guangxi Medical University Cancer Hospital and another 100 healthy women from the First Affiliated Hospital of Guangxi Medical University. The sample collection was approved by the Ethics Committee of the Guangxi Medical University Cancer Hospital. A total of 300 serum samples from OC patients were collected, with a median age of 51 years (17–79 years); 80 cervical cancer patients, with a median age of 50 years (29–71 years); 90 breast cancer patients, with a median age of 51 years (33–71 years); 80 liver cancer patients, with a median age of 52 years (43–62 years); 190 cases of gynecological benign tumor patients, with a median age of 41.5 years (17–66 years); and 200 samples of healthy women, with a median age of 41.5 years (21–70 years) (Table 1). Complete clinicopathological data of all patients were also obtained. A total of 180 normal ovarian tissue samples were obtained from GTEx (https://xenabrowser.net/datapages/), and the ovarian serous cystadenocarcinoma samples were obtained from the TCGA (https://xenabrowser.net/datapages/). The inclusion criteria were as follows: (1) pathologically diagnosed as ovarian cancer and (2) complete survival time and survival outcome. After matching a total of 374 OC samples into the follow‐up study, expression values were transformed to log2 (FPKM + 1).

**TABLE 1:** Summary of serum samples

Type of disease	*N*
Ovarian cancer	300
Epithelial ovarian cancer	251
Serous carcinoma	195
Mucinous carcinoma	40
Endometrioid carcinoma	10
Ovarian clear cell carcinoma	6
Germ cell tumor	9
Sex cord‐stromal tumor	8
Others	32
FIGO stage	
I	61
II	53
III	122
IV	64
Cervical cancer	
FIGO stage	80
I	27
II	24
III	25
IV	4
Breast cancer	
Clinical stage	90
I	20
II	33
III	25
IV	12
Liver cancer	
BCLC stage	80
A	30
B	25
C	25
Gynecological benign tumors	190
Ovarian cyst	33
Mature teratoma of ovary	44
Serous cystadenoma of ovary	26
Mucinous cystadenoma of ovary	10
Uterine fibroids	36
Others	41
Healthy women	200

Abbreviations: BCLC, Barcelona Clinic Liver Cancer; FIGO, International Federation of Gynecology and Obstetrics.

### 2.2 Detection method and model building

In this experiment, the liquid chip technology was prepared by a mixed suspension of fluorescent coded microspheres. After mixing the coded microspheres for different detection substances, a small amount of the test sample was added. The target and cross‐linked molecules on the surface of microspheres precisely bind in the suspension. The two‐color laser is employed to detect red classification fluorescence on microspheres and green reporter fluorescence on the reporter molecule, allowing for the determination of the type and quantity of bound detection substance.

Zhao Bingbing et al.^19^ specifically coated CCL18 and CXCL1 monoclonal antibodies (ab242618, ab206411, Abcam company), as well as C1D, TM4SF1, FXR1, and ZNF573 proteins (homemade) on microspheres (Bio‐Rad company). Biotinylated CCL18 and CXCL1 polyclonal antibodies (ab271200, ab271204, Abcam company), and C1D, TM4SF1, FXR1, and ZNF573 goat anti‐human IgG polyclonal antibodies (ab97225, Abcam company) were used as detection antibodies to determine the content of CCL18 and CXCL1 antigens, as well as C1D, TM4SF1, FXR1, and ZNF573 IgG autoantibodies in serum samples. Antigen and antibody preparation: the expression plasmid (PET‐SUMO plasmid, 5643 bp, Invitrogen Company) encoding C1D, TM4SF1, FXR1, and ZNF573 genes was constructed in a previous study and induced with isopropylthiogalactoside (IPTG, I6758, Sigma company) protein expression. Nickel chelate column affinity chromatography was used to purify the recombinant protein, and SDS‐PAGE was employed to identify the purified protein. The purified C1D, TM4SF1, FXR1, and ZNF573 antigens were ultrafiltered and concentrated using an ultrafiltration tube. A dialysis card was added in phosphate‐buffered saline (PBS) 4°C overnight with stirring. According to biotin labeling kit (11418165001, Thermo company), CCL18, CXCL1 polyclonal antibodies, and C1D, TM4SF1, FXR1, and ZNF573 goat anti‐human IgG polyclonal antibodies labeled biotin. After dialysis, the bicinchoninic acid method (BCA protein quantitative kit, 23227, Thermo company) detects the concentration of each antigen and biotinylated antibody. Verification of the successful coating of CCL18, CXCL1 monoclonal antibodies, and C1D, TM4SF1, FXR1, and ZNF573 antigens on microspheres: According to the operation of microsphere coating kit (Bio‐Rad company) Manual, 1 reaction volume microsphere = 1.25 × 10^6^ microspheres; CCL18 and CXCL1 monoclonal antibodies, as well as C1D, TM4SF1, FXR1, and ZNF573 antigens, are respectively coated with No. 62 and No. 34, and No. 29, No. 37, No. 52, and No. 55 microspheres. A straining buffer was used to dilute the biotinylated secondary antibodies (biotinylated CCL18, CXCL1 polyclonal antibodies, and biotinylated C1D, TM4SF1, FXR1, and ZNF573 goat anti‐human IgG polyclonal antibodies) to 2 μg/ml. About 50 μl of the diluted secondary antibody was added to the experimental wells of 96‐well plate, and 50 μl of straining buffer was added to blank control wells, followed by incubation under shaking for 30 min at room temperature in the dark. The supernatant was aspirated with a wash station, the streptavidin‐phycoerythrin (SPE) was diluted at 1:100, and 50 μl of diluted phycoerythrin was added to each well and incubated at room temperature, followed by supernatant aspiration and buffer refilling. The microspheres were suspended and oscillated for 30 s, and Bio‐plex 200 system (Bio‐Rad company) was employed for liquid suspension chip technology detection. The final mean fluorescence intensity (MFI) value of experimental wells is >2000, proving that microspheres are successfully coated. The value at which MFI value reaches the maximum and tends to the plateau is the optimal concentration of CCL18 and CXCL1 monoclonal antibodies, as well as C1D, TM4SF1, FXR1, and ZNF573 antigen‐coated microspheres. Similarly, biotinylated CCL18 and CXCL1 polyclonal antibodies, as well as the biotinylated C1D, TM4SF1, FXR1, and ZNF573 goat anti‐human IgG polyclonal antibody MFI value reach the maximum, and the detection antibody concentration tending to a plateau phase is the optimal detection antibody concentration. On this basis, a multi‐index joint detection can be successfully established to achieve the best stability liquid suspension chip detection system. Configured standard products: CCL18 and CXCL1 antigen fold dilutions are employed as standards for detecting serum antigens. Serum C1D, TM4SF1, FXR1, and ZNF573 IgG autoantibodies are all tested with IgG protein as standard products. Serum sample detection: the sample is tested using Bio‐plex 200 instrument for liquid suspension chip technology. The specific operation steps are conducted following the operation manual of Bio‐Rad Company. A dose‐response standard curve was drawn to detect CCL18 and CXCL1 antigens, as well as C1D, TM4SF1, FXR1, and ZNF573 IgG‐type autoantibodies using diluted CCL18 and CXCL1 antigens, or IgG protein as the abscissa and MFI value as the ordinate.

One hundred fifty cases of ovarian cancer, 150 cases of benign tumors, and 100 cases of healthy women were used as the model creation group. The logistic regression method was used to establish a diagnostic model for combined detection of serum antigens and antibodies. In addition to the mathematical verification of the model, the remaining 150 patients with ovarian cancer, 40 cases of benign tumors, and 100 healthy women were used as the model verification group to conduct clinical external verification of the constructed diagnostic model.

### 2.3 Follow‐up time

One hundred fifty OC patients from January 2014 to June 2018 whose serum samples were available were followed up by telephone, inpatient and outpatient medical records, etc. The content of the follow‐up included the survival of the patients, and the follow‐up time was as of January 12, 2021. The follow‐up success rate was 80%, and the lost follow‐up rate was 20%. Overall survival (OS) was defined as the last contact time from the first day after surgery to the time of death or last follow‐up.

### 2.4 CIBERSORT score

The 374 cases of ovarian serous cystadenocarcinoma in TCGA were divided into four groups according to the median of CCL18 and TM4SF1 expression levels. The immuneeconv R software package was used for immune scoring, and tumor‐infiltrating immune cells were analyzed in the two groups. The CIBERSORT algorithm was used to explore the distribution difference of immune‐infiltrating cells.^22^

### 2.5 Statistical analysis

Statistical analyses were performed with SPSS 22.0 and R 4.0.3. The distribution of data was skewed and described by the median ± interquartile range. The Wilcoxon rank‐sum test was used to compare two groups, and the Kruskal‐Wallis test was used for multi‐sample group comparisons. Logistic regression was used to construct a combined detection diagnostic model. The count data were expressed as rate, and the chi‐squared test was used for comparison between continuous variables. The receiver‐operating characteristic curve (ROC) was used to compare the diagnostic efficiency. In the survival analysis, patients were divided into high‐expression and low‐expression groups with the median as the cut‐off value. The Kaplan‐Meier method was used for the analysis of survival curves, and the log‐rank test was used to test the difference. The Cox regression model was used for multivariate analysis. A *p*‐value of <0.05 was considered statistically significant.

## 3 RESULTS

### 3.1 Comparison of serum levels of CCL18 and CXCL1 antigens, and C1D, TM4SF1, FXR1, and ZNF573 IgG autoantibodies

**FIGURE 1:**
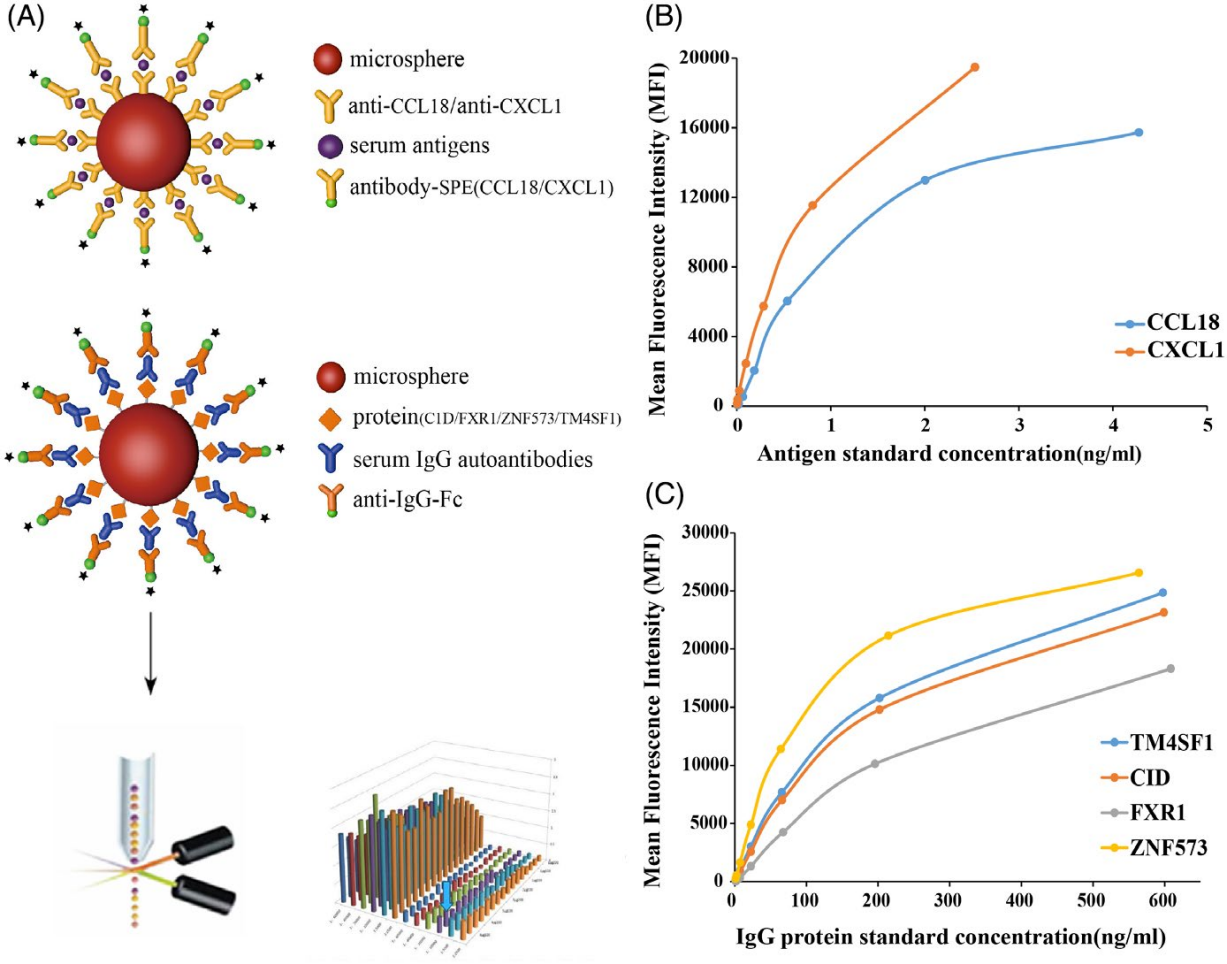
(A) Detection flow chart of the Flow Fluorescence Technology. (B) A dose‐response standard curve of serum CCL18 and CXCL1 antigens detection. (C) A dose‐response standard curve of C1D, TM4SF1, FXR1, and ZNF573 IgG autoantibodies detection

**FIGURE 2:**
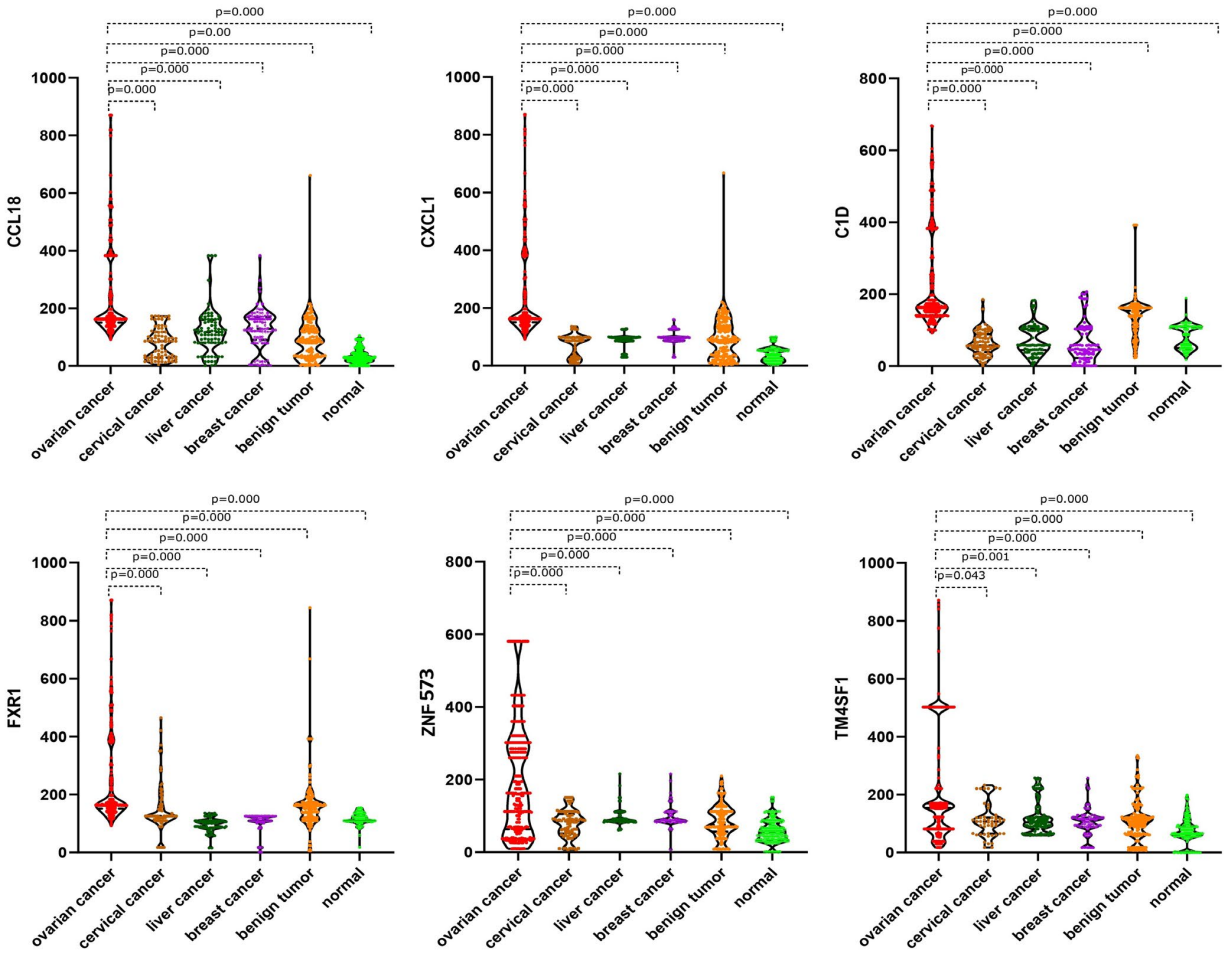
Comparison of serum levels of CCL18 and CXCL1 antigens and C1D, FXR1, ZNF573, and TM4SF1 IgG autoantibodies in ovarian cancer group and other groups

We have listed the detection process of fluorescence analysis technology (Figure 1A). A dose‐response standard curve of was drawn to detect serum CCL18 and CXCL1 antigens (Figure 1B), as well as a dose‐response standard curve of C1D, TM4SF1, FXR1, and ZNF573 IgG protein to detect serum IgG autoantibodies (Figure 1C). After quantitative calculation, the levels of serum CCL18 and CXCL1 antigens, and C1D, TM4SF1, FXR1, and ZNF573 IgG autoantibodies were significantly higher in patients with OC than in those with cervical cancer, liver cancer, breast cancer, gynecological benign tumor patients, and healthy women (Figure 2).

### 3.2 Comparison of the efficacy of individual markers and combined detection models in the diagnosis of OC

Patients with OC (*n* = 300) were regarded as the positive group and those with benign tumors (*n* = 190) and healthy women (*n* = 200) were regarded as the negative control group. The ROC curve of serum antigen and antibody indicators was drawn to detect and diagnose OC. The area under the ROC curve (AUC) of CCL18 and CXCL1 antigens and C1D, FXR1, ZNF573, and TM4SF1 IgG autoantibodies for the diagnosis of OC was 0.944 and 0.939, and 0.847, 0.808, 0.744, and 0.702, respectively (*p* < 0.001). The Youden index was used to determine the optimal cut‐off point. The Youden index was 106.935 μg/L for CCL18, 105.565 ng/L for CXCL1, 120.920 μg/L for C1D, 137.925 μg/L for FXR1, 141.115 μg/L for ZNF573, and 149.055 μg/L for TM4SF1.

**FIGURE 3:**
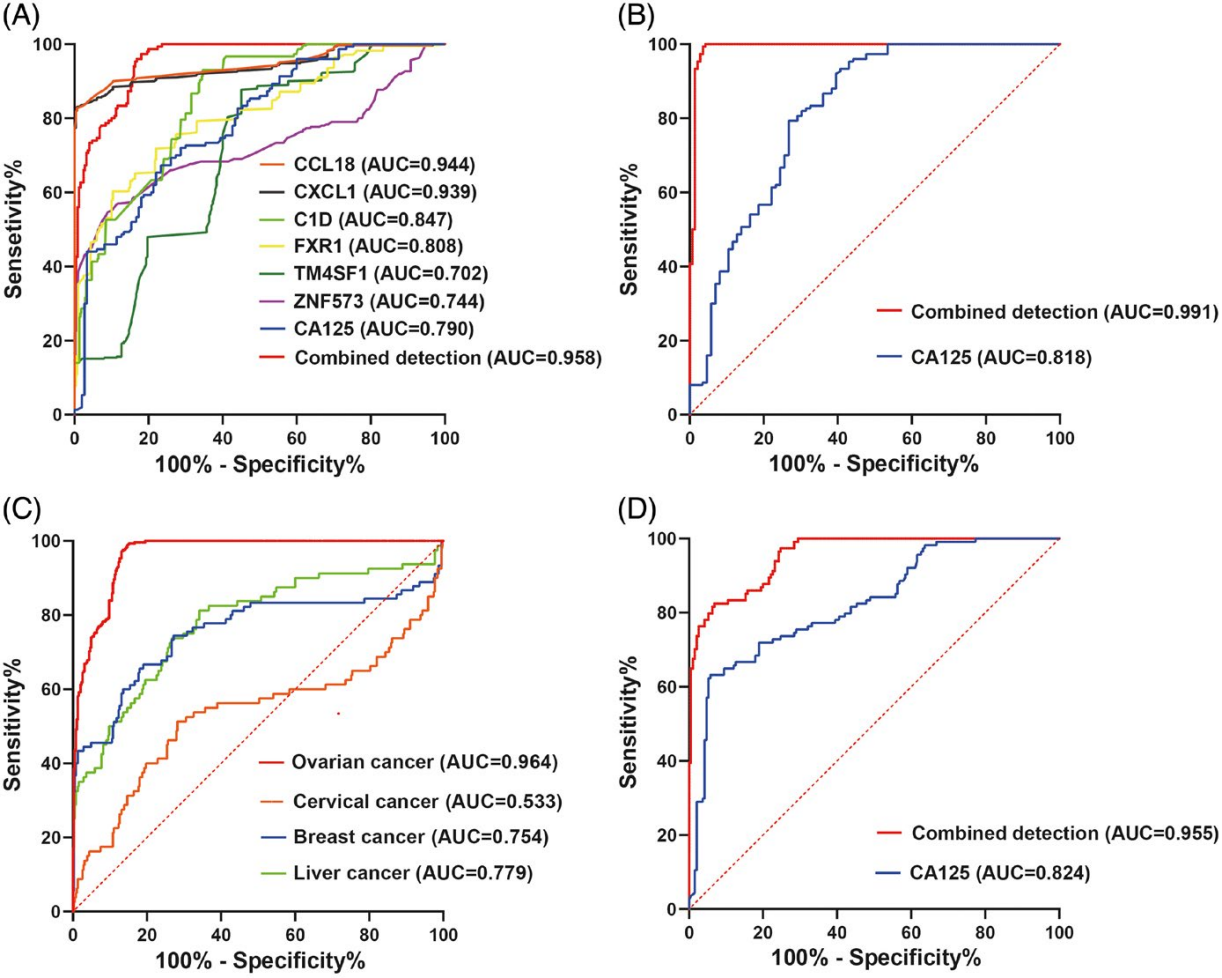
(A) ROC curves of serum CCL18 and CXCL1 antigens and C1D, FXR1, TM4SF1, and ZNF573 IgG autoantibodies for the diagnosis of ovarian cancer in single and combined detection models. (B) ROC curve of the serum antigen‐antibody combined detection model and CA125 alone for diagnosis of ovarian cancer. (C) ROC curve of the serum antigen‐antibody combined detection model for the diagnosis of ovarian cancer and other malignant tumors. (D) ROC curve of the serum antigen‐antibody combined detection model and CA125 alone for diagnosis of early‐stage ovarian cancer

Patients with OC (*n* = 150) were set as the positive group, and those with benign tumors (*n* = 150) and healthy women (*n* = 100) were set as the negative control group. Logistic regression was used to construct the diagnostic model of serum antigen‐ and antibody‐combined detection: Logit(*p*) = −6.244 + 0.014*C1D + 0.005*TM4SF1 + 0.010*ZNF573 − 0.026*FXR1 + 0.072*CCL18 − 0.030*CXCL1 and *p* = exp (−6.244 + 0.014*C1D + 0.005*TM4SF1 + 0.010*ZNF573 − 0.026*FXR1 + 0.072*CCL18 − 0.030*CXCL1)/[1 + exp (−6.244 + 0.014*C1D + 0.005*TM4SF1 + 0.010*ZNF573 − 0.026*FXR1 + 0.072*CCL18 − 0.030*CXCL1)]. The model coefficients passed the test (*p* < 0.001), and the model fit was good (Hosmer and Lemeshow test *p* > 0.05). The ROC curve was constructed (*p* > 0.5 predicts malignant tumors and *p* < 0.5 predicts benign tumors). The AUC for the combined detection of serum antigen and antibody for OC diagnosis was 0.958 (*p* < 0.001), which was higher than the AUC for detection of OC using serum antigen and antibody alone. The ROC curve of serum CA125 was drawn for the same group of samples, and the AUC for the CA125 for OC diagnosis was 0.790 (Figure 3A).

Other patients with OC (*n* = 150), and those with benign tumors (*n *= 40) and healthy women (*n* = 100) for external clinical validation of the model were used. The AUC for the combined detection of serum antigen and antibody for OC diagnosis was 0.991 (*p* < 0.001), which was higher than the AUC for detection of OC using CA125 alone (0.818, *p* < 0.001) (Figure 3B).

### 3.3 Comparison of diagnostic efficacy of combined detection for ovarian cancer and other cancers

The AUC of serum antigen‐antibody combined detection of OC (*n* = 300) was also higher than that of the other malignant tumors, including cervical cancer (*n* = 80), breast cancer (*n* = 90), and liver cancer (*n* = 80), with an AUC of 0.964, 0.533, 0.754, and 0.779, respectively (all *p* < 0.001) (Figure 3C).

The serum antigen‐antibody combined detection model had a positive rate of 77.3% (232/300) in the diagnosis of OC, a negative rate of 83.7% (159/190) in the diagnosis of gynecological benign tumors, and a negative rate of 100.0% (200/200) in healthy controls. The model's diagnostic coincidence rate for OC, gynecological benign tumors, and healthy women was 85.7%, the sensitivity was 77.3%, the specificity was 92.1%, the positive predictive value was 88.2%, and the negative predictive value was 84.1%. The positive rates were 17.5% (14/80), 45.6% (41/90), and 41.3% (33/80) when the model was used for the diagnosis of cervical cancer, breast cancer, and liver cancer, respectively. The results showed that the positive rate, positive predictive value, and positive likelihood ratio of this model were higher for the diagnosis of OC than cervical cancer, breast cancer, and liver cancer (*p* < 0.001) (Table 2).

**TABLE 2:** Comparison of the diagnostic efficiency of the serum antigen‐antibody combined detection model for the diagnosis of ovarian cancer and other malignant tumors

	Positive/*N*	Positive rate	Positive predictive value	Positive likelihood ratio	χ^2^	*p*
Ovarian cancer	232/300	77.3%	88.2%	9.723	—	—
Cervical cancer	14/80	17.5%	31.1%	2.001	99.047	0.000
Breast cancer	41/90	45.6%	56.9%	5.736	33.291	0.000
Liver cancer	33/80	41.3%	51.6%	5.195	38.964	0.000

Note

*p*‐values represent the comparison of the positive rate of ovarian cancer and cervical cancer; the comparison of the positive rate of ovarian cancer and breast cancer; and the comparison of the positive rate of ovarian cancer and liver cancer.

### 3.4 Comparison of the diagnostic efficacy of serum antigen‐antibody combined detection model and CA125 alone in the detection of early‐stage OC (stage Ⅰ‐Ⅱ)

The staging of OC was based on the 2012 standard of the International Federation of Obstetrics and Gynecology (FIGO). A total of 114 cases of early‐stage OC (stage Ⅰ to Ⅱ) were regarded as the positive group and 190 cases of benign tumors were regarded as the control group. The ROC curve was drawn, and the serum antigen‐antibody combined detection model was established. The AUC for the diagnosis of early‐stage OC was 0.955 (*p* < 0.001), which was higher than that of CA125 alone (0.824, *p* < 0.001) (Figure 3D). The sensitivity and specificity of the two methods for diagnosing early‐stage OC were 86.0% and 75.4%, respectively, and the specificity for diagnosing benign tumors was 83.7% and 67.9%, respectively. The results indicated that the combined detection of serum antigen and antibody was significantly better than CA125 alone in the diagnosis of early‐stage OC (*p* < 0.001).

### 3.5 Effects of serum CCL18 and CXCL1 antigens, and C1D, TM4SF1, FXR1, and ZNF573 IgG autoantibodies on the prognosis of OC patients

**FIGURE 4:**
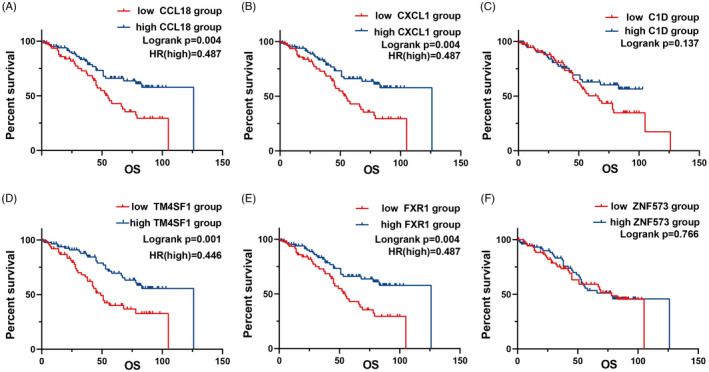
Survival curves of serum (A) CCL18 antigen, (B) CXCL1 antigen, (C) C1D IgG autoantibodies, (D) FXR1 IgG autoantibodies, (E) TM4SF1 IgG autoantibodies, and (F) ZNF573 IgG autoantibodies in patients with ovarian cancer

The serum levels of CCL18 and CXCL1 antigen, and C1D, TM4SF1, FXR1, and ZNF573 IgG autoantibodies in OC patients were divided into two groups with the median of each group as the cut‐off value. The Kaplan‐Meier analysis showed that the 5‐year OS rates were 78.2%, and the median OS time was 73 months. The median OS of the high CCL18 expression group (*p* = 0.004) and the high CXCL1 expression group (*p* = 0.004) was significantly higher than that of their corresponding low‐expression groups. Similarly, the median OS of the high FXR1 expression group (*p* = 0.004) and the high TM4SF1 expression group (*p* = 0.001) was significantly higher than that of their corresponding low‐expression groups (Figure 4A–F). The COX regression univariate analysis showed that CCL18, CXCL1, FXR1, and TM4SF1 were the key factors influencing the OS time of patients with OC (*p* < 0.01), whereas the COX multivariate analysis showed that CCL18 (HR: 0.537, *p* = 0.019) and TM4SF1 (HR: 0.480, *p* = 0.006) may be independent protective factors affecting the OS time of OC patients (Table 3).

**TABLE 3:** COX univariate and multivariate analyses of the overall survival time of patients with ovarian cancer

	Univariate analysis			multi‐factor analysis		
	HR	95% CI	*p*	HR	95% CI	*p*
CCL18 (µg/L)	0.480	0.287–0.804	0.005	0.537	0.319–0.905	0.019
CXCL1 (ng/L)	0.480	0.287–0.804	0.005			
C1D (µg/L)	0.675	0.400–1.139	0.141			
FXR1 (µg/L)	0.480	0.287–0.804	0.005			
TM4SF1 (µg/L)	0.435	0.259–0.733	0.002	0.480	0.284–0.812	0.006
ZNF573 (µg/L)	0.926	0.555–1.542	0.767			

### 3.6 Differences in the expression of CCL18, CXCL1, C1D, TM4SF1, FXR1, and ZNF573 in normal ovarian tissues and OC tissues

**FIGURE 5:**
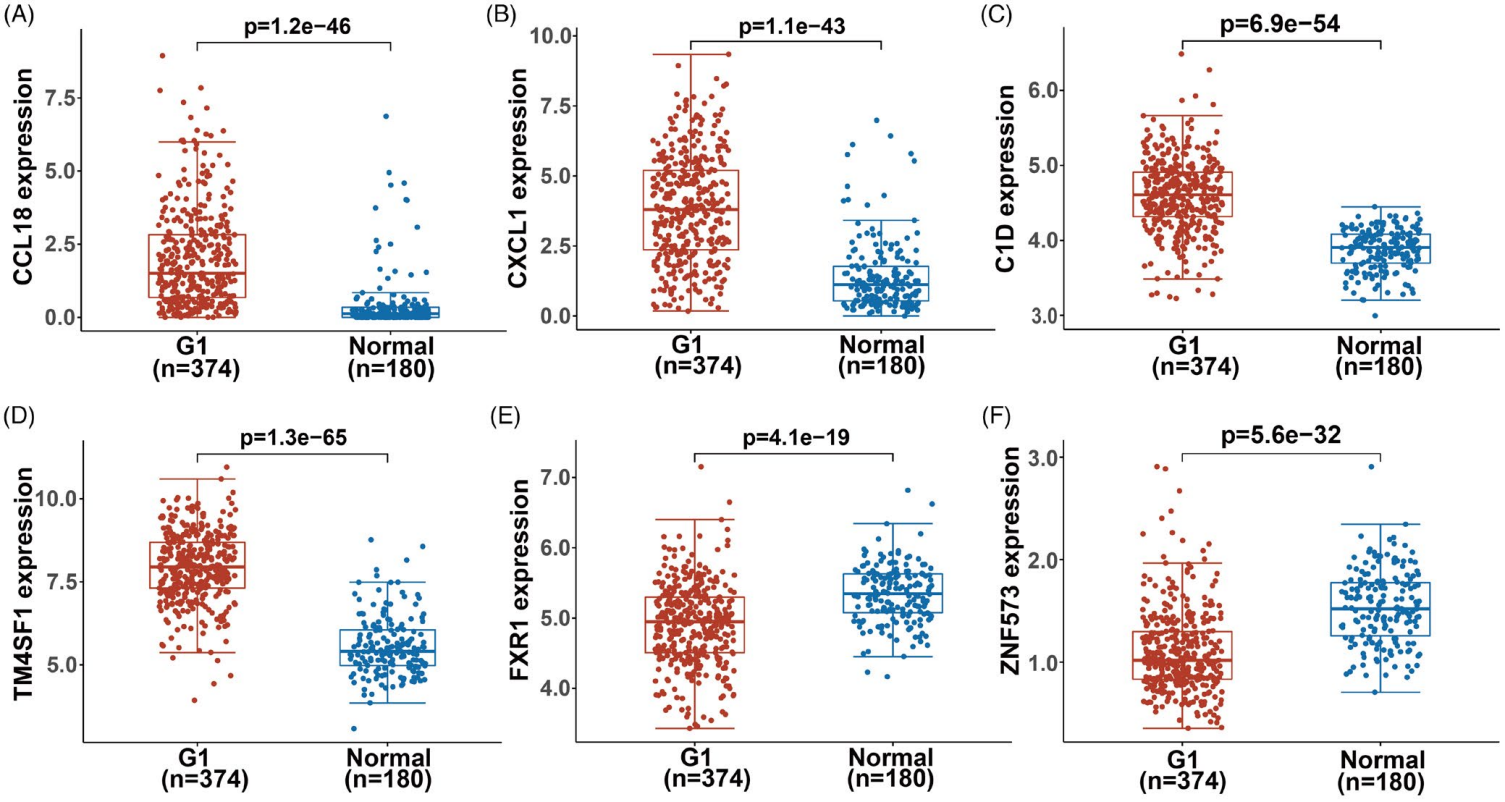
Comparison of the expression of (A) CCL18, (B) CXCL1, (C) C1D, (D) TM4SF1, (E) FXR1, and (F) ZNF573 in ovarian cancer and normal tissues in TCGA and GTEx databases

RNA sequencing data of 374 patients with ovarian serous cystadenocarcinoma were downloaded from the TCGA database through the Genomic Data Commons (GDC) data portal. The data of 180 normal tissue samples were obtained from GTEx (V8, https://gtexportal.org/home/datasets). All data were standardized to transcripts per million (TPM). Analysis of differential gene expression showed that CCL18, CXCL1, TM4SF1, C1D, FXR1, and ZNF573 were highly expressed in OC tissues compared with normal tissues (Figure 5A–F).

### 3.7 Relationship between the expression of CCL18, CXCL1, C1D, FXR1, TM4SF1, and ZNF573 in OC tissues and OS time

**FIGURE 6:**
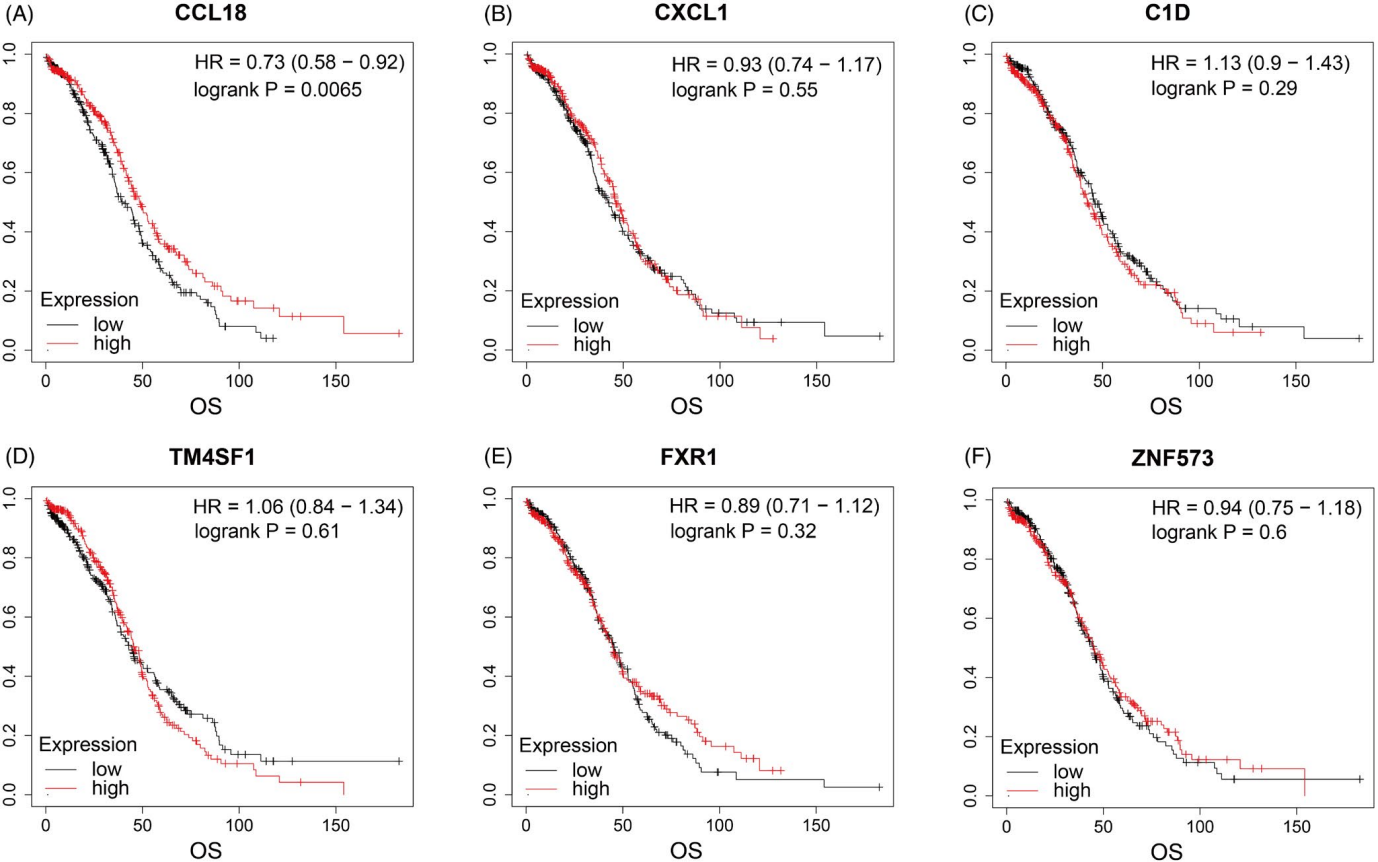
Survival curve of (A) CCL18, (B) CXCL1, (C) C1D, (D) FXR1, (E) TM4SF1, and (F) ZNF573 in ovarian cancer tissues

RNA sequencing data and corresponding clinical information of 557 cases of OC were obtained from TCGA, and an online Kaplan‐Meier plotter (http://kmplot.com/analysis/) was used to predict survival. Gene expression was grouped by the median, and the Kaplan‐Meier curve showed that the median OS of the CCL18 high‐expression group was higher than that of the low‐expression group (HR = 0.73, *p* = 0.006), while CXCL1, C1D, TM4SF1, ZNF573, and FXR1were no difference in the survival curve (*p* > 0.05) (Figure 6A–F).

### 3.8 Relationship between the expression of CCL18 and TM4SF1 and immune cell infiltration

**FIGURE 7:**
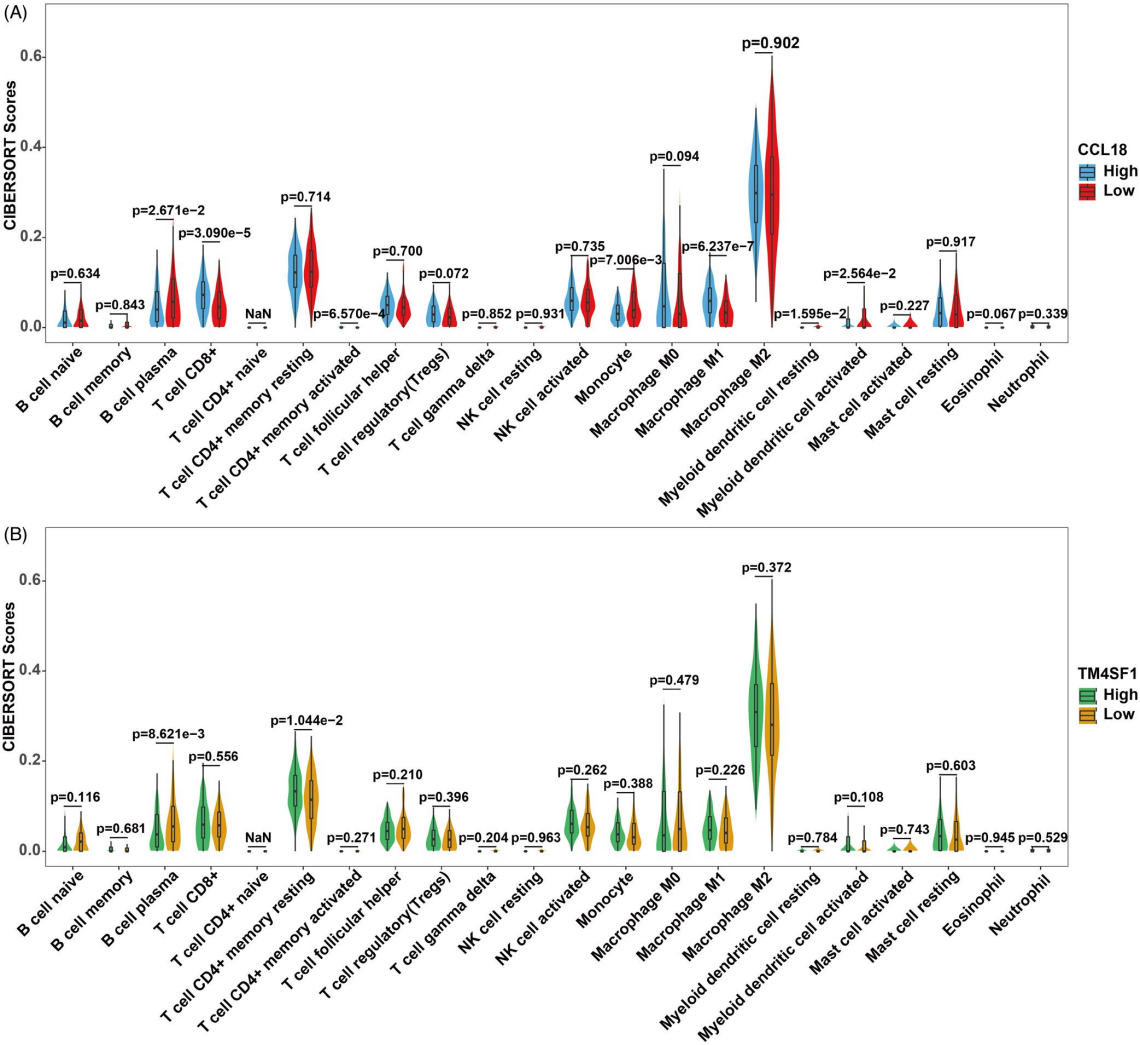
Violin image of immune cell infiltration. (A) Violin image of immune cell infiltration in the CCL18 group. (B) Violin image of immune cell infiltration in the TM4SF1 group

The 374 cases of OC tissues obtained from TCGA were divided into high‐expression groups and low‐expression groups according to the median of CCL18 and TM4SF1 expression as the cut‐off value. The CIBERSORT algorithm was utilized to analyze the difference in the distribution of tumor‐infiltrating immune cells between the two groups (Figure 7). There was substantial infiltration of M2 macrophages (0.297 ± 0.12%), resting memory CD4+ T cells (0.13 ± 0.06%), CD8+ T cells (0.058 ± 0.064%), and plasma cells (0.045 ± 0.036%) in OC tissues. The degree of infiltration of CD8+ T cells and M1 macrophages was significantly higher in the high CCL18 expression group compared with the low‐expression group (*p* < 0.001). However, the degree of infiltration of monocytes was significantly lower in the high CCL18 expression group compared to that in the low‐expression group (*p* < 0.05) (Figure 7A). The degree of plasma cell infiltration was significantly lower in the high TM4SF1 expression group than in the low‐expression group (*p* < 0.001), while the degree of infiltration of resting memory CD4+ T cells was higher in the high‐expression group than in the low‐expression group (*p* < 0.05) (Figure 7B).

## 4 DISCUSSION

Ovarian cancer is the fifth most common cause of cancer‐related deaths in women.^23^ The high mortality rate may be due to the insidious onset of the disease at an early stage, and only a small number of early‐stage patients may be successfully detected through conventional testing methods. In recent years, the systemic inflammatory response has been proven to be one of the important factors that promote tumor occurrence, development, invasion, and metastasis, as well as drug resistance. Inflammatory cells and inflammatory factors can induce gene mutations, change the tumor microenvironment, and regulate the immune system to promote tumor development.^24^ Therefore, inflammatory factors could be used as novel diagnostic and predictive biomarkers for OC. Preliminary studies in our laboratory indicated that the combined detection of CCL18 and CXCL1 chemokines and C1D, FXR1, TM4SF1, and ZNF573 autoantibodies has great potential for early diagnosis of OC.^20^, ^21^

Nuclear nucleic acid–binding protein C1D plays a role in multiple cellular processes, transcriptional regulation, genome stability monitoring, DNA repair, and RNA processing, all of which are necessary to maintain the life cycle of the host.^25^ When the tumor microenvironment changes, C1D induces the production of autoantibodies in the serum of patients with epithelial ovarian cancer (EOC). The production of autoantibodies may be due to the ectopic expression of C1D, which stimulates the immune system.^26^, ^27^ Fragile X mental retardation syndrome–related protein 1 (FXR1) has been confirmed to be amplified in lung cancer, breast cancer, head and neck cancer, and OC.^28^, ^29^ Its overexpression is related to cell growth, migration, and invasion. The down‐regulation of the zinc finger protein 573 (ZNF573) gene can promote the growth of OC cells.^30^ According to previous clinical studies, the expression level of ZNF573 protein in the serum of patients with ovarian malignant tumors was significantly higher than that of patients with benign ovarian tumors and healthy individuals.^31^ C‐X‐C motif chemokine ligand 1 (CXCL1) is associated with increased cell transformation, tumor growth, and invasive potential. CXCL1 overexpression promoted proliferation, while CXCL1 silencing inhibited proliferation in EOC cells.^32^ Transmembrane 4 L Six Family Member 1 (TM4SF1) is related to tumor metastasis and regulates angiogenesis, making it a potential target for anti‐angiogenesis and anti‐tumor therapy.^33^ Gao et al.^34^ found that TM4SF1 was up‐regulated in non–small cell lung cancer tissues and cell lines and was closely related to survival time, tumor size, lymph node metastasis, distant metastasis, and clinical staging. Similarly, the positive expression rate of TM4SF1 protein in OC tissues was higher than that of benign ovarian tumors and normal ovarian epithelial tissues, which may be associated with the abnormal proliferation of ovarian epithelial cells and malignant transformation. C‐C Motif Chemokine Ligand 18 (CCL18) is mainly produced by tumor‐associated macrophages, which may be associated with tumor cell migration.^35^ Schutyser et al.^36^ determined that the expression level of CCL18 in patients with EOC and ascites was higher than that of patients with benign ovarian tumors with high immunostaining in the interstitial area and a limited number of CCL18 antibodies.

Based on the influence of inflammatory factors on EOC, we used a liquid chip system for joint detection. The liquid chip contains various microspheres with different fluorescent dyes, which are combined with corresponding serum antigens and antibodies in a covalently cross‐linked manner. The analyte and the fluorescent‐encoded microspheres are mixed to form an immune complex, which can be detected by dual‐laser detection of fluorescent signals to simultaneously identify different molecules in the same sample.^37^ The present study combined different types of markers for early and differential diagnosis of OC. The results showed that the combined detection of serum antigen and antibody can improve the specificity and sensitivity of OC diagnosis, and the diagnostic efficiency was higher than that of other epithelial malignancies.

Tumor cells exist in the complex tumor microenvironment, which is necessary for tumor growth and survival. Several non‐tumor cell types, including tumor‐infiltrating leukocytes (TIL), constitute tumor stroma. Immune infiltration is usually a heterogeneous mixture of immune cells, including innate and adaptive immune populations, as well as active (such as cytotoxic T lymphocytes) and inhibitory (such as regulatory T cells and bone marrow–derived suppressor cells) immune cells and functionally related cell types. The importance of TIL varies with cancer histology. The presence of certain immune subgroups usually shows a beneficial prognostic effect in one type of malignant tumor and an adverse effect in another type of cancer. With the development of new immunotherapeutics aimed at targeting these cells, the importance of TIL assessment continues to increase. Recent studies have found that T lymphocyte subsets (such as CD8+) can predict response to existing and emerging immunotherapies, highlighting the importance of studying tumor‐associated immune cells as potential predictive biomarkers.^38^, ^39^, ^40^ CCL18 is a small (7.8 kDa) inflammatory protein, also known as dendritic cell–chemokine 1 (DC‐CK1) or pulmonary and activation‐regulated chemokine (PARC).^41^, ^42^ It is a chemotactic cytokine expressed by various lymphocytes. Previous studies have shown that CCL18 overexpression may be associated with tumor growth and development, especially in the tumor microenvironment, and it is considered a useful marker for diagnosis and prognosis.^36^, ^43^ The main targets of CCL18 include helper T lymphocytes (CD4+), cytotoxic T lymphocytes (CD8+), naive T lymphocytes (CD45RA+), and B lymphocytes. Leung et al.^43^ analyzed 89 gastric cancer patients and found that high expression of CCL18 significantly correlated with OS and disease‐free survival (DFS). In addition, the results of multivariate analysis of OS and DFS showed that CCL18 and tumor stages were independent prognostic factors, which are consistent with our current prognostic results on OC. Studies have shown that CCL18 is involved in the immune response of T and B cells. The prognostic significance of T cell infiltration in OC has been confirmed by numerous studies. T lymphocyte infiltration in OC is associated with a good prognosis.^44^ Moreover, immunohistochemical analysis of M1 and M2 macrophages in OC showed that patients with a higher ratio of M1/M2 macrophages had a better prognosis, indicating that macrophages play an important role in the progression of OC.^45^ In the current study, we used the CIBERSORT algorithm to determine the proportion of infiltrating immune cells and found higher infiltration of CD8+ T cells and M1 type macrophages in the ovarian serous cystadenocarcinoma tissues in TCGA data in the high CCL18 expression group compared with their corresponding low‐expression groups. This may be because the prognosis of the high CCL18 expression group appeared slightly better than that of the low CCL18 expression group. Based on findings from previous studies and our observations on the improved prognosis of OC in the high‐expressing CCL18 group and the low‐expressing CCL18 group, it is reasonable to assume that the expression of CCL18 in the tumor environment may attract and activate immune cells, thereby enhancing the immune response against malignant tumors. Therefore, CCL18 may also be an independent prognostic factor for survival in OC.

Although TM4SF1 may be a key factor influencing the prognosis of OC in our study, Yang and colleagues found that the positive expression of TM4SF1 protein was not an independent factor for prognosis.^34^ Qiang et al.^46^ showed that the expression of TM4SF1 in colorectal cancer tissues was significantly higher than that in non‐tumor tissues, which was positively correlated with poor prognosis. In the analysis of immune infiltrating cells, there was almost no difference in the distribution of immune infiltrating cells between different groups of TM4SF1. The impact of TM4SF1 on the prognosis of OC should be further verified with a large sample size. TM4SF1 has been recently confirmed to be associated with various tumors, including colorectal cancer, OC, lung cancer, etc. and could be a potential factor in the treatment of OC in the future.^47^

This study has several limitations. Firstly, it is a single‐region retrospective study with relatively small sample size. Secondly, the time of chemotherapy was not strictly controlled in the samples collected in this study. Different regimens of chemotherapy may change the immune microenvironment of the patient's body, leading to errors in detection. Further studies should be conducted to consider this aspect. In addition, the relationship between CCL18 expression and prognosis in OC tissues extracted from databases such as TCGA requires further clinical verification. Therefore, large‐scale and multi‐center prospective studies are needed to further verify the independent prognostic significance of CCL18 in OC.

In summary, combined detection of CCL18 and CXCL1 chemokines, and C1D, TM4SF1, FXR1, and ZNF573 autoantibodies can improve the specificity and sensitivity of OC diagnosis, and its diagnostic efficiency is higher than that of other malignant tumors. CCL18 may serve as a novel biomarker for the early diagnosis and prognosis of OC. Although these possibilities require further verification, they still provide new avenues for the future clinical diagnosis and prognosis of OC.

## CONFLICT OF INTEREST

The authors declare no potential conflicts of interest regarding research, authorship, and/or publication of this article.

## ETHICS STATEMENT

The ethical review was approved by the Institute Ethics Committee of Guangxi Medical University Cancer Hospital (LW2021094) and individual consent for this retrospective analysis was waived.

## Data Availability

The datasets generated during and/or analyzed during the current study are available from the corresponding author on reasonable request.
